# Coping styles in substance use disorder (SUD) patients with and without co-occurring attention deficit/hyperactivity disorder (ADHD) or autism spectrum disorder (ASD)

**DOI:** 10.1186/s12888-015-0530-x

**Published:** 2015-07-14

**Authors:** Linda M. Kronenberg, Peter J.J. Goossens, Jooske van Busschbach, Theo van Achterberg, Wim van den Brink

**Affiliations:** 1 Department of residency training MANP mental health, Dimence, P.O. Box 5003, 7400 GC Deventer, The Netherlands; 2 Dimence Group, Center for Mental Health Care, Expertise Centre Developmental Disorders, Deventer, The Netherlands; 3 Dimence Group, Center for Mental Health Care, Expertise Centre Bipolar Disorders, Deventer, The Netherlands; 4 Radboud University Medical Center, Radboud Institute for Health Sciences, IQ healthcare, Nijmegen, The Netherlands; 5 Department of Public Health, University Centre for Nursing and Midwifery, Faculty of Medicine and Health Sciences, Ghent University, Ghent, Belgium; 6 GGZ-VS, Institute for Education of Clinical Nurse Specialist in Mental Health, Utrecht, The Netherlands; 7 University of Groningen, University Medical Centre Groningen, Groningen, The Netherlands; 8 Centre for Health Services and Nursing Research, KU Leuven, Leuven, Belgium; 9 Department of Public health and Caring Sciences, Uppsala University, Uppsala, Sweden; 10 Amsterdam Institute for Addiction Research, Academic Medical Center University of Amsterdam, Amsterdam, The Netherlands

**Keywords:** Coping styles, Substance Use Disorder, Attention Deficit/Hyperactivity Disorder, Autism Spectrum Disorder

## Abstract

**Background:**

Patients with a substance use disorder (SUD) and co-occurring attention deficit hyperactivity disorder (ADHD) or autism spectrum disorder (ASD) often start using substances in an attempt to cope with the stress related to their ADHD or ASD. To improve treatment for these patient groups, it is important to identify and compare the various coping styles between SUD patients with and without ADHD or ASD and with subjects from a general population sample.

**Methods:**

Cross-sectional study using the Utrecht Coping List (UCL) in 50 SUD patients, 41 SUD + ADHD patients, 31 SUD + ASD patients and 1,200 railway employees.

**Results:**

Compared with the reference group, all three SUD groups showed a significant higher mean on the Palliative reaction, Avoidance, and Passive reaction subscales of the UCL. The scores for all UCL subscales of the SUD and the SUD + ADHD groups were very similar. However, the SUD + ASD group scored higher on Passive reaction and lower on Reassuring thoughts than the SUD and the SUD + ADHD groups and lower on Expression of emotions subscale in comparison with the SUD + ADHD group.

**Conclusions:**

Regardless of the presence of a co-occurring disorder, SUD patients reported more palliative, avoidant and passive coping when confronted than people in the general population. In addition, SUD patients with co-occurring ASD were different from other SUD patients in their coping and professionals should take this into account when working on more adaptive coping strategies with these patients.

## Background

There are many reasons for why people start using substances, continue to use them and eventually become dependent on them. Speaking broadly, three core reasons for substance use can be distinguished: (a) positive reinforcement (i.e. instant pleasure and euphoria provided by substance use); (b) negative reinforcement (i.e. instant relief from feelings of anxiety, depression or insecurity provided by substance use and thus self-medication); and (c) habitual/compulsive substance use (i.e. substance use is no longer associated with positive or negative reinforcement, but has become an automatic behaviour (e.g. chain smoking) [1–7].

People with Attention Deficit/Hyperactivity Disorder (ADHD) are more likely to use substances and become addicted because they are either looking for thrills (i.e., positive reinforcement) or seeking relief from their ADHD symptoms (i.e., negative reinforcement) [8, 9]. Cannabis, for example, may be used to cope with insomnia; stimulants may be used to cope with hyperactivity and the inattention which this brings with it. In contrast, individuals with an Autism Spectrum Disorder (ASD) have been shown to mostly use substances to suppress social anxiety, make it easier to get in contact with their surroundings and reduce stress (i.e., negative reinforcement) [10].

In a recent study, we have shown that patients with a substance use disorder (SUD) and co-occurring ADHD or ASD *initially* started to use alcohol and/or drugs to cope with ADHD and ASD associated stress. Substance use may initially ameliorate the symptoms and the related stress but it may worsen the situation later [10]. An important question is whether this pattern of substance use is specific for the the different groups or part of a more general way of dealing with stressful situations (i.e., part of a general coping strategy).

### Classifications of coping strategies and styles

Coping refers to the cognitive and behavioural efforts of individuals to manage their internal and external demands which are appraised as taxing or exceeding the resources of the individual [11]. People can differ with respect to the general or dominant strategy which they use to deal with stressful situations and a variety of coping styles have been identified. In a recent review, Nielsen and Knardahl [12] identified the following - partly overlapping - coping styles: problem-focused versus emotion-focused coping [13], active versus passive coping [14, 15], adaptive versus maladaptive coping [16], and engagement versus disengagement coping [17].

Unfortunately, there is no consensus on the classification of coping styles or the best model of human coping to be used [17]. For example, in a two-year prospective sample of 3,738 working adults, Nielsen and Knardahl [12] found that the use of what can be considered *dysfunctional* coping strategies was related to poor mental health while the use of what could be considered *functional* coping strategies was related to good mental health. Specific coping strategies and overall coping styles showed *some* stability but were nevertheless open to change over time and thus malleable.

Research has shown that ADHD patients use more confrontational and escape-avoidance behaviours for the management of stressful situations and less planned problem-solving strategies when compared to a control group of individuals without ADHD [18, 19]. However, in a different study, ADHD patients *also* used ‘positive reappraisal’ as an adaptive coping strategy intended to give some positive meaning to the stressful situation via a focus on personal growth and learning [19]. For ASD patients, a disengagement coping style has been found to be associated with significantly higher levels of both behaviour and emotional problems [20]. Finally, people with a substance use disorder (SUD) reported more disengagement and avoidance behaviours than other coping behaviours [21–23].

To our knowledge, however, no studies have compared the coping styles of SUD patients with and without a co-occurring ADHD (SUD + ADHD) or co-occurring ASD (SUD + ASD). It can thus be asked (1) what coping styles are displayed by adult SUD patients with and without co-occurring ADHD or ASD and (2) whether significant differences emerge when the coping styles of these groups of SUD patients are compared to those of a reference group of individuals from the general population.

### Hypotheses derived from the available literature

Based on the literature summarized before, we hypothesize that SUD + ADHD patients will report more confronting behaviour and more positive reappraisal than SUD and SUD + ASD patients. In contrast, SUD + ASD patients will report more disengagement than SUD+ ADHD and SUD patients. We also hypothesize that SUD + ADHD patients will report more avoidance behaviour than SUD and SUD + ASD patients. Finally, we expect all of the patients to score similarly for socialization behaviours despite stronger limitations in the social interaction and communication capacities of ASD patients.

To test these hypotheses, coping style data of SUD patients with and without a co-occurring ADHD or ASD were compared to each other and to existing coping style data for a general population reference group.

## Methods

### Study design

A cross-sectional study was conducted using the Utrecht Coping List (UCL) in SUD patients with and without a comorbid ADHD or ASD and a group of healthy controls.

### Participants

The patient population consisted of SUD patients without and with a comorbid ADHD (SUD + ADHD) or comorbid ASD (SUD + ASD). Inclusion criteria: inpatients and outpatients seeking treatment for SUD; age 18–65; IQ >80; DSM-IV diagnosis of SUD with or without DSM-IV diagnosis of adult ADHD or ASD; and mastery of the Dutch language. Exclusion criteria: somatic complaints not directly related to SUD. All 122 patients also participated in the study previously reported by Kronenberg et al. [10]. Although ASD and ADHD appear to frequently co-occur with each other [24, 25], the DSM-IV does not allow a diagnosis of ADHD and a diagnosis of ASD at the same time. In the present research, only nine patients were diagnosed with SUD and both ADHD and ASD but excluded from the analyses.

The general population reference group consisted of 1,200 Dutch railway employees who were very similar to the patient groups in age (mean 43 years) and gender (5% females) [26].

A certified medical ethics committee (Commissie Mensgebonden Onderzoek Regio Arnhem-Nijmegen) and the institutional review board of Dimence (Commissie Wetenschappelijk Onderzoek) approved the study. All patients received both oral and written information about the study and signed an informed consent form.

### Assessment

In order to identify coping styles, we administered the Utrecht Coping List (UCL) to all patients. The UCL consists of 47 items constituting seven subscales: (1) Active problem solving (ACT; confronting, employment of purposeful problem-solving strategies), (2) Palliative reaction (PAL; try to feel better by smoking, drinking, distraction of problems, relaxing), (3) Avoidance (AVOI; avoid situation, waiting, keeping clear of the problem), (4) Socialization (SOC; seeking comfort from other or asking for help), (5) Passive reaction (PAS; rumination, drawing back, retreat, pondering, incapacity to do something about the situation), (6) Expression emotions (EXP; expression of annoyance or anger, letting off of steam), (7) Reassuring thoughts (REA; calms oneself by thinking that worse things can happen, self-encouragement).

All UCL items are rated on a four-point Likert scale ranging from 1 (=never) to 4(=very often). A higher score on a coping style thus indicates that this response style is used more often. Table 1 shows that the internal consistency of the UCL subscales in the reference group was good (Cronbach’s alpha > .70) for most scales and acceptable (Cronbach’s alpha .50-.70) for the EXP-scale.

**Table 1:** Reliability (Cronbach’s alphas) for UCL-scales in the SUD group (n = 122) and the reference group (n = 1,200)

	ACT	PAL	AVOI	SOC	PAS	EXP	REA
	7 items	8 items	8 items	6 items	7 items	3 items	5 items
Reference group	.82	.76	.73	.75	.70	.64	.70
Research group	.77	.58	.71	.83	.70	.65	.58

**Table 2:** Demographic and clinical characteristics of the research and reference groups

	Ref. Group	SUD	SUD + ASD	SUD + ADHD
N	1200	50	31	41
Male (%)	95.6	72	94	68
Age mean	43	46	40	37
*Living (%)*				
Alone	9	38	52	37
With parents		16	10	5
With partner		28	12	17
With partner and children	88	12	7	29
With children alone		-	-	5
Protected living		-	19	2
Otherwise	3	6	-	5
*Income (%)*				
Employed	100	30	26	39
Partner, family, friends		12	3	2
Social welfare unemployment		28	29	27
Social welfare; declared unfit to work		26	42	27
Otherwise		4	-	5
*Education (%)*	not applicable			
None		8	3	7
Primary Education		24	36	44
Secondary Education		35	39	35
Highschool		28	16	15
University		6	7	-
*Type of treatment (%)*	not applicable			
None		8	5	12
Outpatient detox		2	-	-
Clinical detox		6	3	12
Outpatient_ drug free treatment		58	65	59
Clinical_ drug free treatment		10	3	5
Daycare		14	-	2
Mental health hospital		2	7	-
Otherwise		-	7	7
*Primary drugs (%)*	no information			
Complete remission		4	3	2
Alcohol		66	71	39
Heroin		4	-	2
Cocaine		8	7	7
Amphetamines		-	3	15
Cannabis		18	13	24
More than one		-	3	10

In the SUD groups, the internal consistency of the subscales was found to be good for four scales (ACT, AVOI, SOC, and PAS) and acceptable for three scales (PAL, EXP and REA).

### Statistical analyses

The UCL scores were normally distributed for all groups. A series of one-way ANOVAs (*p* < .05) was conducted on the UCL scores to test for significant differences between the three groups of patients. An additional series of ANCOVAs was performed to control for group differences in the living situations of the patients. Post-hoc independent T-tests (*p* < .05) were conducted to clarify the differences between the groups when significant differences were revealed by the ANOVAs or ANCOVAs. In addition, the standardized effect sizes (Cohen’s *d*) were calculated to facilitate the interpretation of significant outcomes: *d* < 0.20 no relevant difference; *d* =0.20–0.30 small difference; *d* = 0.30–0.80 moderate difference; and *d* > 0.80 large difference [27]. In order to prevent false negative findings in this exploratory study, we decided not to correct the significance level for multiple comparison.

## Results

A total of 122 patients participated in the study: 50 patients with SUD only, 41 patients with SUD + ADHD and 31 patients with SUD + ASD. Most of these patients were in a substance abuse treatment programme at the time of assessment although a small percentage (13%) was not. There were substantial group differences in the living conditions of the patients compared to each other and compared to the healthy controls. There were also substantial differences between the three patient groups in the substances which were used (for example: amphetamines F3.413/*p* = 0.036 and cannabis F 4.684/*p* = 0.011). Based on these findings, the SUD group comparisons were adjusted for differences in living conditions (*p* = 0.025). We decided not to adjust for the substances that were used, because this could result in overcorrection and produce false negative findings.

On average, the interviews with the patients lasted 15 min. For the ASD group, it took a bit more time to complete the UCL interview on average.

**Fig. 1:**
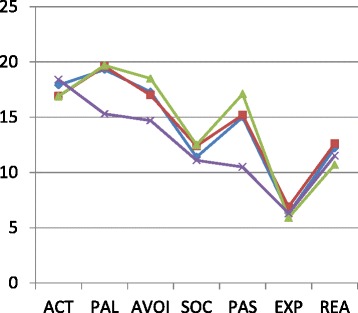
The mean score of coping style for three patient groups and one reference group.  SUD,  SUD + ADHD,  SUD + ASD,  Reference

Figure 1 shows the similarities and differences in the coping styles of the four groups at a glance .

Table 3 shows significant differences in coping between the three patient groups, but the differences were limited to the coping styles of *Passive reaction* and *Reassuring thoughts* (ANOVA B-C-D). Similar findings were found when differences in the living situations of the patients were controlled for (i.e. living alone or not) (ANCOVA B-C-D). In particular, SUD + ASD patients reported more *Passive reaction* and used less *Reassuring thoughts* than both the SUD group (d = −0.534 and d = 0.560) and the SUD + ADHD group (d = 0.511 and d = −0.713). In addition, the SUD + ASD group reported less *Expression of emotions* than the SUD + ADHD group (d = 0.511). No significant differences were observed between the SUD and the SUD + ADHD group.

**Table 3:** Overview of differences in coping behaviours

	Ref.groep -A-Mean/sd	SUD -B-Mean/sd	SUD + ADHD -C-Mean/sd	SUD + ASD -D-Mean/sd	A-B		A-C		A-D		B-C		B-D		C-D		Anova;B-C-D groups	Ancova;B-C-D groups	
	N = 1200	N = 50	N = 41	N = 31	t-toets	cohen d	t-toets	cohen d	t-toets	cohen d	t-toets	cohen d	t-toets	cohen d	t-toets	cohen d		f	sign.
ACT	18.40/3.58	17.86/3.56	16.93/4.05	16.87/3.66	.289	0.151	.025*	0.388	.029*	0.426	.245	0.245	.235	0.277	.949	−0.016	.387	1.1	.35
PAL	15.32/3.62	19.34/2.89	19.60/3.71	19.70/3.68	.000**	−1 .227	.000**	−1.167	.000**	−1.200	.698	−0.081	.628	−0.109	.919	0.024	.880	.13	.88
AVOI	14.71/3.29	17.30/3.69	16.98/3.62	18.47/4.26	.000**	−0.741	.000**	−0.655	.000**	−0.987	.675	0.089	.2	−0.293	.116	0.377	.246	1.3	.28
SOC	11.07/2.95	11.44/3.38	12.43/3.80	12.54/3.51	.442	−0.117	.026*	−0.403	.03*	−0.451	.188	−0.278	.171	−0.317	.915	0.026	.288	1.3	.28
PAS	10.55/2.67	14.98/3.71	15.17/3.27	17.10/4.22	.000**	−1.335	.000**	−1.502	.000**	−1.815	.798	−0.055	.022*	−0.534	.033*	0.511	.036	3.1	.05
EXP	6.25/1.70	6.26/1.95	6.85/1.74	5.93/2.12	.097	−0.005	.032*	−0.351	.419	0.165	.133	−0.322	.484	0.161	.049*	0.511	.121	2	.14
REA	11.54/2.57	12.18/2.54	12.63/2.72	10.73/2.61	.081	−0.251	.014**	−0.414	.101	0.311	.413	−0.172	.017*	0.560	.004**	−0.713	.010	5	.008

* = *P* < 0.05

** = *p* < 0.01

From Table 3 and Figure 1, it can be seen that all patient groups showed large differences from the reference group: compared to the reference group, patients showed (much) more *Passive reaction* (SUD d = −1.335, SUD + ADHD d = −1.502, SUD + ASD d = −1.815), more *Palliative reaction* (SUD d = −1.227, SUD + ADHD d = −1.167, SUD + ASD d = −1.200) and more *Avoidance* (SUD d = −0.741, SUD + ADHD d = −0.655, SUD + ASD d = −0.987).

## Summary and discussion

Compared to the reference group, all three SUD groups scores much higher on the *Palliative reaction*, *Avoidance* and *Passive reaction* subscales*.* The scores for the SUD and SUD + ADHD groups are very similar on all UCL subscales. However, the SUD + ASD group scores are significantly higher on *Passive reaction* and lower on *Reassuring thoughts* compared to the SUD and SUD + ADHD groups and lower on *Expression of emotions* compared to the SUD + ADHD group.

These findings only partly supported our first hypothesis, namely that patients with SUD + ADHD would show more confronting behaviour but also more positive reappraisal than patients with SUD and SUD + ASD. The patients with SUD + ADHD in our study did not show more *Active problem solving* behaviour than the other groups while their average score on *Reassuring thoughts* was higher than in the SUD + ASD group but not the SUD group.

Our second hypothesis was confirmed: patients with SUD + ASD in our study reported more disengagement behaviour compared to the patients with only SUD and those with SUD + ADHD. The SUD + ASD patients scored higher on *Passive reaction* and lower on *Expression of emotions* than the SUD + ADHD patients.

Our third hypothesis that the SUD + ADHD group would report more avoidance behaviour than both the SUD and SUD + ASD groups was not supported. All three patient groups scored higher on *Avoidance* than the reference group with no significant differences detected between the three patient groups.

Our fourth and final hypothesis that all patient groups would score similarly with respect to socialization behaviours (i.e.seeking comfort from other or asking for help) was supported: all patient groups scored similar with no significant between group differences.

Our initial hypotheses were derived from a very small body of studies involving mostly patients with ADHD or ASD and not from studies with SUD patients with a comorbid ADHD or ASD. Data from patients with a single diagnosis obviously cannot be applied automatically to patients with co-occurring diagnoses [28]. It is certainly possible that the symptoms and coping styles associated with specific disorders will interact and affect each other. For example, the confronting behaviour which we expected to see in patients with ADHD can conceivably be counteracted by palliative behaviour, avoidance behaviour or passive behaviour on the part of patients with SUD *and* ADHD . This would then explain why the SUD + ADHD group did not score higher than the other patients groups and reference group on confronting behaviour.

In contrast, it was expected that the SUD + ADHD group would show avoidance as a particular coping style compared to the SUD and SUD + ASD groups since studies on both the single diagnosis of SUD [21–23] and the single diagnosis of ADHD [18, 19] reported that both patient groups used more avoidance coping, but this turned out not to be the case.

As expected, the patient groups scored similarly on socialization behaviours despite the often strong limitations on the social interaction and skills of patients with SUD + ASD. Substance use, particularly for the patients with ASD, may have facilitated social interaction [10, 29].

The results of the current study confirm previous results showing passive disengagement coping strategies to be associated with high levels mental health problems [30]. However, the causal nature and exact direction of this association, if causal, has yet to be determined. It is certainly possible that passive disengagement and mental health problems are causally related, but it is also possible that the two simply represent different aspects of the same condition. If the two are causally related, moreover, it is not yet clear if passive disengagement as a coping style can lead to mental health problems or mental health problems can lead to the increased use of passive engagement as a coping strategy. Additional research is clearly needed.

### Study strengths, limitations and future research

The present study has both strengths and limitations. The most important strengths are the relatively large subgroups of patients included in the study and the availability of a relatively well-matched reference group. A first limitation is the relatively low internal consistency of some of the UCL subscales in the patient group. This is probably due to some of the patients and especially those in the SUD + ASD group having difficulties distinguishing between the ‘sometimes’ versus ‘often’ response options. Research assistants were available to assist but may not have been approached. A second limitation is that comorbid conditions other than ADHD and ASD were not identified and utilized as an exclusion criterion. A third limitation is that we did not take into account the primary substance of abuse as a confounder for the differences in coping style between the three patient groups, because this may have lead to overcorrection and false negative findings. However, it can not be excluded that differences in coping style between the patient groups are partly explained by differences in the different patterns of alcohol and drug use. A fourth limitation is that patient groups differed considerably in their living conditions. However, statistical adjustment for these differences did not change the findings. In order to prevent false negative findings in this relatively small study, we decided not to correct for multiple comparisons. A fifth limitation is that males were highly overrepresented in all three patients groups and in the reference group. We therefore cannot extrapolate from the current findings to draw conclusions about female patients with SUD, SUD + ADHD or SUD + ASD. This is important, because it is also well known that males and females differ considerably in coping strategies [26]. Finally, we would like to note that this study did not include patients with only ADHD or ASD and therefore we have no information about the influence of SUD on the coping style of patients with ADHD and ASD.

### Practice implications

Overall, the preferred coping styles for *all* SUD groups were *Palliative reaction*, *Avoidance* and *Passive reaction*. These coping styles may be effective in the short run because they decrease the jumble of emotions and mixture of agitation, distress and anxiety which can plague these patients [10]. In the long run, however, the use of a *palliative, avoidance or passive* coping style can interfere with the ability of patients to effectively deal with their impairments, anticipate possible problems and handle problems when they occur. Guiding patients towards a more active, adaptive and problem-focused manner of coping should be at least one goal of treatment.

In addition, total abstinence or reduced consumption should be a treatment goal for all patients with a combination of disorders. In SUD + ADHD patients, substance use may hinder *Active problem solving* and *Reassuring thoughts*. For SUD + ASD patients, reducing their substance use might even be the primary treatment goal as substance use is known to increase passivity. However, in this patient group substance use may also enable socialization. The care professional might collaborate with the patient on the development of a relapse prevention plan, for example, and help the patient to anticipate problems and thus prevent them or be better prepared to deal with them in a functional as opposed to dysfunctional manner.

## Conclusions

SUD patients in this study used more *Palliative reaction*, *Avoidance* and *Passive reaction* coping styles when confronted with unpleasant events and problems than the general population. In addition, patients with SUD + ASD used more *Passive reaction* and less *Reassuring thoughts* than patients with SUD or SUD + ADHD. These findings are important for understanding SUD patients in general and those with a comorbid diagnosis in particular. Patients with SUD + ADHD frequently want to become totally abstinent because their SUD *exacerbates* their ADHD symptoms and lead to major problems in many domains of adult life. In contrast, patients with SUD + ASD often only want to reduce their drinking, because it can *facilitate* their social interaction.
